# Time-dependent blood eosinophilia count increases the risk of kidney allograft rejection

**DOI:** 10.1016/j.ebiom.2021.103645

**Published:** 2021-10-20

**Authors:** Luc Colas, Linh Bui, Clarisse Kerleau, Mohamed Lemdani, Karine Autain-Renaudin, Antoine Magnan, Magali Giral, Sophie Brouard

**Affiliations:** a INSERM, CHU Nantes, Nantes Université, Centre de Recherche en Transplantation et Immunologie UMR1064, Centre Hospitalier Universitaire de Nantes, ITUN 30 bd Jean Monnet, Nantes 44093, France; b Centre Hospitalier de Mouscron, Belgique, Service de néphrologie, Belgium; c Service de Néphrologie-Immunologie Clinique, CHU Nantes, Nantes Université, Nantes, France; d Département of Biomathematiques, Faculté de Pharmacie and Biologie, Université de Lille, Lille, France; e Département d'anatomie et Cytologie Pathologique, CHU Nantes, Nantes Université, Nantes, France; f Université de Versailles Saint-Quentin Paris-Saclay, Hôpital Foch, INRAe UMR 0892, Paris, Suresnes, France; g Labex IGO, F-44000 Nantes, France.; h Centre d'Investigation Clinique en Biothérapie, Institut de Transplantation Urology and Nephrology (ITUN), Centre Hospitalier Universitaire de Nantes, 30 bd Jean Monnet, Nantes 44093, France

**Keywords:** Eosinophilia, Donor-specific antibodies (DSAs), Graft rejection, Type 2 inflammation, ABMR, antibody-mediated rejection, BCEo, blood count eosinophils, CCL-X, chemokine n°X, CNI, calcineurin inhibitor, COPD, chronic obstructive pulmonary disease, DAMP, danger-associated molecular pattern, DSA, donor-specific antibody, DSAdn, *de novo* donor-specific antibody, ECD, extended criteria donor, eGFR, estimated glomerular filtration rate, HLA, human leukocyte antigen, HPR, histology proven rejection, HR, hazard ratio, IFN-x, interferon n°X, IgX, immunoglobulin of isotype X, IL-X, interleukin n°X, IS, immunosuppresssive, KTR, kidney transplant recipient, LTR, lung transplant recipient, MHC, major histocompatibility complex, OCS, oral corticosteroid, PTLD, posttransplant lymphomatous disease, TCMR, T-Cell mediated rejection, TSLP, thymic stromal lymphopoietin, VCAM-x, vascular cell adhesion molecule n°x

## Abstract

**Background:**

Growing evidence suggest that type 2 immune effectors play a role in solid organ transplantation. The aim of this study was to evaluate the impact of blood count eosinophils (BCEo) on immunological outcomes in kidney transplant recipients with stable graft function after 3 months post-transplant.

**Method:**

We performed cause-specific Cox model considering BCEo, the use of calcineurin inhibitors and systemic corticoids as time-dependent explicative variables on a prospective cohort of 1013 kidney transplant patients who experienced kidney allograft rejection and/or the appearance of *de novo* donor specific antibodies after excluding common causes of increased BCEo..

**Findings:**

BCEo ≥ 0.3 G/L was associated with a 3-fold increased risk of rejection independent of immunosuppressive regimen after 3 months post-transplant in patients without pre-transplant DSAs and with CNI-based immunosuppression. No association between BCEo either with donor specific antibodies or graft survival was noticed.

**Interpretation:**

These observations in this large cohort support the hypothesis of eosinophils in allo-immunity in human and claim for further mechanistic research.

**Funding:**

This study was supported by the French National Research Agency, The “Institut de Recherche en Santé Respiratoire des Pays de la Loire” and the University hospital of Nantes.

**Research in Context**

**Evidence before this study**

Two recent studies have highlighted the role of IgE in transplant rejection strongly suggesting that type 2 immunity effectors such as eosinophils could have a role in solid organ transplantation immunopathology. We searched in Pubmed, Embase, Web of Science relevant articles encompassing the following terms: “eosinophilia”, “transplant rejection”, “graft rejection”, “kidney transplantation”, “kidney graft rejection”. Mechanistic studies in mice model of transplantation (skin and heart) showed a cytotoxic role of eosinophil participating in acute and chronic rejection. In human, whereas the role of eosinophils in transplantation remains unclear, some case reports and/or studies in a few patients have shown a positive correlation between eosinophilia and solid organ rejection a few days before it occurs. In this study, we evaluated the association of time-dependent variation in blood count of eosinophils (BCEo) and allograft rejection and the appearance of *de novo* anti-HLA donor-specific antibodies (DSAdns)) in a prospective cohort of 1013 kidney recipients with stable renal function at 3 months post-transplant.

**Added value of this study**

Our data thus revealed that a BCEo ≥ 0.3 G/L threshold could be an interesting and routine biological marker for monitoring immunological outcomes along with other routine parameter such as DSAdn in kidney transplant recipients without pre-transplant DSAs and with CNI-based immunosuppression at steady state (i.e., 3 months post-transplant).

**Implications of all the available evidence**

In clinical practice after eliminating common causes of an increase in BCEo (PTLD, acute allergic process, parasitic or viral infections, BCEo ≥ 0.3 G/L could lead to monitor more regularly biological parameters associated with rejection such as DSAdn (IgG anti-HLA class I or II with MFI > 2000) and/or proteinuria > 1 g/24 h and/or hematuria (> 10 red blood cells/mL) and/or an increase of 25% in serum creatinine compared to baseline. Though, multicentric studies challenging BCEo > 0.3 G/L threshold but also evaluating the optimal time points of BCEo titration are needed. At last, these observations open new perspectives and directions that raise the question of the involvement of eosinophils and type 2 immunity in kidney allograft rejection.

Alt-text: Unlabelled box

## 1 Introduction

Type 2 inflammation is mainly characterized by a high rate of IL-4, IL-5 and IL-13 secretion leading to IgE synthesis, an increase in blood count eosinophils (BCEo) and eosinophil and mast cell/basophil tissue infiltration. Type 2 inflammation is mostly associated with parasitic infection and atopic/allergic diseases [1]. There is growing evidence that type 2 inflammation may play a role in autoimmune disorders, particularly in systemic erythematous lupus, where specific IgE against double-stranded DNA is associated with a severe phenotype of kidney and pulmonary injuries in mice and humans [2], [3], [4]. In allotransplantation, IgE against donor major histocompatibility complex (MHC)is also associated with acute graft rejection in mouse models of heart and skin transplantation [5], thus suggesting a role for effector cells such as mast cells and/or eosinophils.

The implication of eosinophils in acute and chronic rejection has been shown in an experimental mouse model to depend on the cytotoxic effect of cationic and basic proteins released after eosinophil degranulation in the graft [6]. In cardiac and skin allograft models, type 2 cytokines such as IL-5, IL-4 and IL-13 seem to play central roles. IL-5 inhibition *in vivo* results in a drastic decrease in eosinophil infiltrate and in complete or partial rejection inhibition in cardiac and skin graft models [7], [8], [9]. IL-4 and IL-13 also participate in allograft rejection via the upregulation of adhesion molecules such as VCAM1, which is mainly present on the eosinophil membrane [10], and the increase in eotaxin secretion by endothelial cells, which is crucial for eosinophil diapedesis in synergy with IL-5 [11,12]. Altogether, these observations suggest that activated eosinophils infiltrating allografts can induce graft injuries and dysfunction in a type 2 inflammation dependent manner.

In humans, whereas the role of eosinophils in transplantation remains unclear, some case reports and/or studies in a few patients have shown a positive correlation between eosinophil infiltrate in graft biopsies and acute rejection in cardiac, lung, liver and kidney transplantation models, which is often associated with an increase in relative blood BCEo (percentage of total leukocytes) a few days prior, suggesting that blood eosinophilia is predictive of an immunological event occurring in the graft [13], [14], [15], [16], [17], [18]. In the present study, we evaluated the association of time-dependent variation in blood count of eosinophils (BCEo) and allograft rejection and the appearance of *de novo* anti-HLA donor-specific antibodies (DSAdns) in a prospective cohort of 1013 kidney recipients with stable renal function at 3 months post-transplant.

## 2 Methods

### 2.1 Selection of the study population

**Fig. 1:**
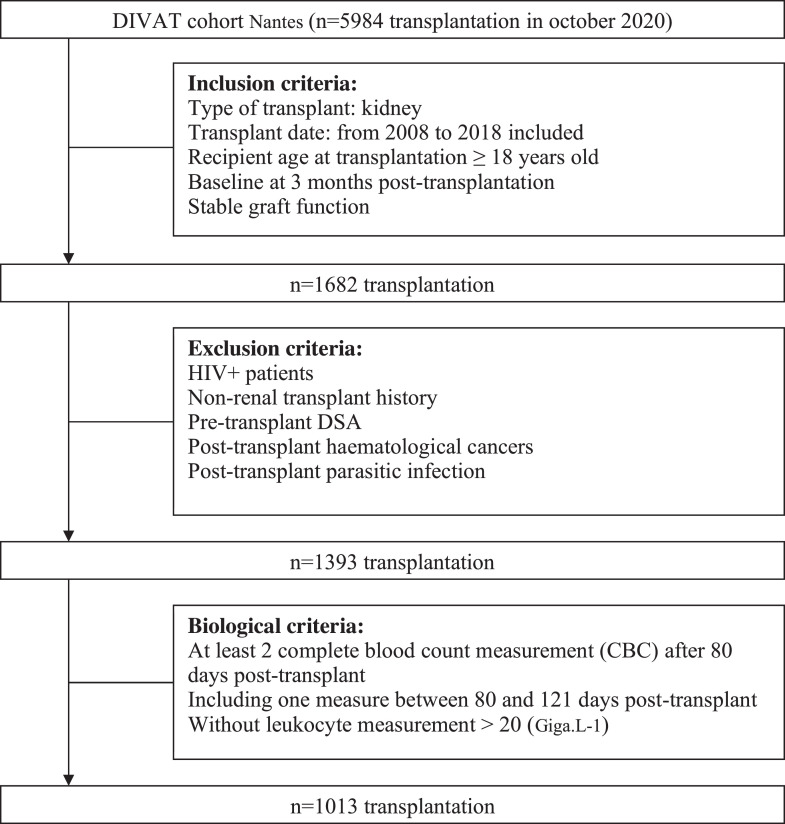
Flowchart of the study.

Daily practice clinical and biological data were extracted from the prospective cohort of kidney transplant recipients (KTRs) [DIVAT cohort from Nantes University Hospital (Données Informatisées et VAlidées en Transplantation) (www.divat.fr, French Research Ministry: RC12_0452, last agreement No. 13 334, No. CNIL for the cohort: 891735)]. First, a total of 5984 KTRs with medical follow-up in Nantes University Hospital were screened. Adult (≥ 18-year-old) KTRs alive between January 2008 and December 2018 with stable graft function at 3 months post-transplant were selected (*n* = 1682). Stable graft function at 3 months post-transplant was defined as creatinine < 150 µmol/L and proteinuria < 1 g/24 h under standard immunosuppression (calcineurin inhibitors (CNIs) or mTOR inhibitors and antimetabolite +/- corticosteroids). We established a baseline period at 3 months post-transplant to obtain the steady state of BCEo. Indeed, high-dose of systemic corticosteroids (intra-venous or oral), immunosuppressive drugs (CNI, antimetabolites, mTOR inhibitors) and induction therapy are given within the first 3 months after transplantation and the impact of immunosuppressive drugs (CNI and oral corticoids mostly) combination on BCEo is yet not well established. Among those 1682 KTRs, patients with pretransplant DSA or who had undergone simultaneous kidney-pancreas transplantation were excluded. Patients with post-transplant haematologic disorders, active parasitic infection and HIV infection were also excluded since those factors are known to increase BCEo (*n* = 1393) [19]. At last, Patients with a lymphocyte count greater than 20 G/L or patients who did not have eosinophil count data in the baseline period (3 months post-transplant +/-10 days) and during the follow-up period (2 BCEo at least) were excluded leading to a total of 1013 KTRs enrolled in the study. The follow-up period began at 3 months post-transplant, and all patients were administratively censored in December 2018 (Fig. 1).

### 2.2 Clinical and biological data selection

Classical risk factors likely to influence graft immunological outcomes were extracted from the database. Donor features included age, sex, living or deceased donor status, cold ischemia time and extended criteria donor (ECD) data [20,21]. The recipient characteristics were age, sex, induction therapy (depleting vs non-depleting), initial and actual maintenance therapy at eosinophil count, renal replacement therapy before transplantation, initial nephropathy, baseline 3 months post-transplant estimated glomerular filtration rate (eGFR) assessed by the MDRD formula, and previous HLA immunization status. The transplantation parameters were the rank of transplantation (*i.e.* number of kidney transplantation) and the number of HLA-A/B/DR mismatches. As the baseline period began at 3 months post-transplant, the previous immunization status was defined as positive if a rejection episode or HLA antibody (IgG) was registered before the baseline period. All the rejection episodes were histology proven rejection (HPR). HPR was divided into 3 subsets according to the Banff consensus: [1] antibody-mediated rejection (ABMR), [2] *T*-cell-mediated rejection (TCMR) and [3] borderline rejection [22]. HLA and DSA antibody detection were based on high-resolution bead-based assay (One Lambda®, Thermo Fisher Scientific, California, USA).

Routine blood eosinophil counts after 3 months post-transplant and all subsequent counts using automated flow cytometry (morphological gating on eosinophils) were noted. All eosinophil counts in this study refer to blood eosinophil counts. Classical and available risk factors likely to influence blood eosinophil count changes were extracted from the database. HIV-positive patients, patients diagnosed with post-transplant lymphoproliferative disorders (PTLDs), active parasitic infections or acute allergic process which could cause an increase in BCEo [19], were excluded.

The appearance of kidney allograft rejection (humoral or cellular rejection including borderline rejection) and the appearance of *de novo* DSA with time-dependent variation in BCEo were used to define analysis outcomes.

### 2.3 Histological analysis of kidney transplant biopsy

Surveillance kidney transplant biopsies were routinely taken during patient follow-up at 3 months post-transplant for 50.2% of the KTR and for 52.9% KTR at 12 months post-transplant. For cause kidney transplant biopsies were also taken during follow-up either in case of a serum creatinine increase of 25% serum creatinine baseline or proteinuria ≥ 1 g/24 h or hematuria (10 red blood cell/mL) or DSAdn against class I and/or class II HLA of IgG isotype with MFI ≥ 2000. Kidney transplant biopsies were carried out by experimented nephrologists with a biopsy gun (Bard Biopsy, Tempe, Arizona, USA). The biopsies were immediately fixed in 10% formalin and subsequently embedded in paraffin, and the produced slides were stained with hematoxylin and eosin (HE), May Grünwald Giemsa (MGG) and Masson trichrome. All kidney transplant biopsies were analyzed according to the Banff classification [22] by an anatomopathologist specialized in nephropathology. The presence of myeloid cells (neutrophils, eosinophils, and basophils) was quantified as the percentage of the total surface if present. All percentage comparisons were performed using a statistical independence test “Chi2 test” under R software version 4.0.3.

### 2.4 Ethical statement

This study was performed in accordance with the Declaration of Helsinki and approved by the National French Ethics Committee (DIVAT (Données Informatisées et VAlidées en Transplantation) (www.divat.fr, French Research Ministry: RC12_0452, last agreement No. 13 334, No. CNIL for the cohort: 891735)). All participants enrolled in this study signed informed consent forms.

### 2.5 Statistical analysis

The analysis was performed in 2 steps. Step 1 was an explanatory variable reduction process based on clinical ground and statistical methods. The principal explanatory variable of interest was multiple eosinophil counts during follow-up. All other predictors of the survival outcome in the raw dataset were selected by 2 independent nephrologists. Variables with small variance and/or redundant variables (checked by using the variance inflation factor or exploratory multidimensional data analysis) were removed. Ten predictors were included in the multivariable regression model: ECD, recipient features (age, sex, multiple BCEo, and the use of corticosteroids and/or CNIs as maintenance therapy at each BCEo), previous immunization status (presence of rejection episodes, HLA antibody or DSAdn antibody before the 3-month post-transplant baseline eosinophil count) and the rank of transplantation. Step 2 involved a multivariable regression model to investigate the etiologic relationship between the dynamic eosinophil count during follow-up and kidney allograft rejection and DSAdn appearance. The outcomes were survival data with multiple changes updated during the patient follow-up. We used a time-dependent covariate cause-specific Cox regression model (competitive risk model for aetiologic purpose) [23,24]. Three explicative variables were considered time-dependent covariates: BCEo and the use of corticosteroids and/or CNIs as maintenance therapy at each BCEo measurement to overcome the uncertainties of the association of these two immunosuppressive drugs on BCEo. To obtain the cause-specific hazard ratio, we studied kidney allograft rejection appearance (DSAdn, death and graft failure defined as return to dialysis or pre-emptive retransplantation were censored) based on the time between the baseline BCEo and the occurrence of HPR. We also studied DSAdn appearance based on the time between the baseline eosinophil count 3 months post-transplant and the occurrence of DSAdn (HPR, death and graft failure defined as return to dialysis or pre-emptive retransplantation, were censored). Then, we explored the relationship between graft failure based on the time between the baseline eosinophil count 3 months post-transplant and the occurrence of either return to dialysis or pre-emptive retransplantation (HPR, death and DSA appearance were censored). At last, we studied the association between the severity of HPR and BCEo in KTR who experienced rejection.

The proportional hazards assumption and the log-linearity assumption of the Cox model were evaluated by Schoenfeld and martingale residuals, respectively [25], [26], [27]. As the log-linearity of the hazard ratio was not confirmed with the quantitative eosinophil count variable, it was thus modeled as a categorical variable. We defined the *a priori* relevant blood eosinophil threshold based on available data mostly from [1] severe eosinophilic asthma and anti-IL-5 monoclonal therapeutic antibodies [28,29] and [2] the lower limit of detection of eosinophil count by automated fluorescence flow cytometry as greater than 0.03 G/L [30], and [3] the within-subject biological variability as up to 20% [31] or variability under corticosteroid therapy (inverse correlation between the OCS dose and BCEo) [32]. Taking into account those parameters, we defined *a priori* threshold of 0.3 Giga/L (equivalent to 300 eosinophils/mL and corresponding to a relative BCEo of approximately 4% assuming a total leukocyte count of 7 G/L) was thus used to categorize BCEo defined as follows: high (≥ 0.3 Giga/L) or low (< 0.3 Giga/L). This *a priori* threshold was confirmed fisrt by inspecting Martingale residuals according to continuous blood count eosinophils, then by plotting the spline function of blood eosinophil according to hazarad ratio using cause-specific multivariable Cox model (Figs. S1A and B) and at last by performing time-dependent multivariable analysis of immunological event onset (rejection and/or DSA) in KTRs during follow-up according to BCEo as categorical variable (0-0.1; 0.1-0.2; 0.2-0.3; > 0.3) (Table S1). The statistical significance (alpha risk) was set at 0.05. All analyses were performed using *R* software version 4.0.3 using Thernau et al package (https://github.com/therneau/survival).

### 2.6 Role of funding source

None of the funder had any role in the present study.

## 3 Results

### 3.1 Demographic characteristics of the kidney transplant population

The demographic characteristics of the patients are described in Tables 1 and 2. The median follow-up period was 78 months post-transplant (3–100 months). After 3 months post-transplant, 75 patients experienced their first rejection episode during follow-up: 75 patients had HPR (29 ABMR, 29 TCMR, and 16 borderline rejection). Rejection episodes appeared at a median time of 13.3 months (2.7–116 months). Ninety-eight (98) patients experienced DSAdn during follow-up. Among them, 24 patients experienced a rejection episode (15 ABMR, 7 TCMR, and 2 borderline rejections). DSAdn appeared at a median time of 24.5 months (3–110 months). All immunological events occurred after at least 2 BCEo measurements. Most patients had eosinophil measurements every 3 months in the first 5 years of follow-up.

**Table 1:** Description of recipient, donor, and transplantation characteristics of the global study population (qualitative variables).

Characteristics	Missing data		*N* = 1013	
	*n*	%	*n*	%
**RECIPIENT**				
Male gender	-	-	631	62.3
Rank of the graft ≥ 2	-	-	188	18.6
Initial nephropathy: Glomerulonephritis	-	-	273	26.9
Pyelonephritis			471	46.5
Vascular nephropathy			107	10.6
Diabetic nephropathy			69	6.8
Other disease and unknown			93	9.2
Replacement therapy: Pre-emptive transplantation	-	-	232	22.9
Peritoneal dialysis			129	12.7
Hemodialysis			652	64.4
**DONOR**				
Deceased donor	-	-	859	84.8
ECD	-	-	428	49.8
**TREATMENTS**				
Maintenance immunosuppressive by CNI	-	-	1009	99.6
Maintenance immunosuppressive by mTOR inhibitor	-	-	13	1.3
Maintenance immunosuppressive by MMF/MPA/AZA	-	-	1002	98.9
Maintenance immunosuppressive by Corticosteroids	-	-	867	85.6
Induction: No induction	-	-	15	1.5
Non depletant therapy			511	50.4
Depletant therapy			487	48.1
**IMMUNOLOGY**				
Number of HLA-A/B/DR mismatches >4	-	-	201	19.8
Positive anti-class I HLA antibody	97	9.6	294	32.1
Positive anti-class II HLA antibody	107	10.6	234	25.8
Rejection in the first three months post-transplantation	-	-	53	5.2
Rejection after three months post-transplantation (biopsy)	1	0.01	75	7.4
ABMR	-	-	29	2.9
TCRM	-	-	29	2.9
Borderline	-	-	16	1.6
DSA de novo in the first three months post-transplantation	27	2.7	26	2.6
DSA de novo after three months post-transplantation	-	-	98	9.7

**Table 2:** Description of recipient, donor, and transplantation characteristics of the global study population (quantitative variables).

Characteristics	Missing data		*N* = 1013			
	n	%	Mean	SD	Min	Max
Recipient age (years)	-	-	54.1	14.1	18	87
Cold ischemia time (hour)	-	-	13.9	8	0.6	42.7
3 month serum creatinine (μmol.L^−1^)	13	1.3	152.8	64.1	51	764
3 month eGFR MDRD (ml.min^−1^)	13	1.3	46.1	17.8	6.1	132.4
1st measure of number of circulating eosinophils (Giga.L^−1^)	-	-	0.1	0.09	0	1.27

### 3.2 Higher eosinophil count during follow-up was associated with a high risk of a rejection episode

**Fig. 2:**
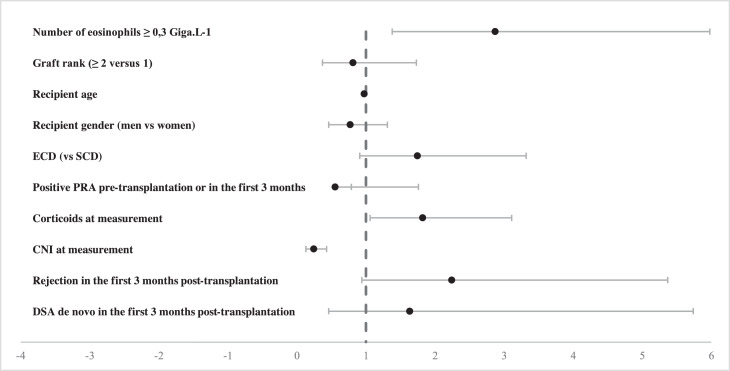
Forest plot of the time-dependent multivariable analysis of the first kidney allograft rejection episode (*n* = 75) in KTRs (*n* = 1013) during follow-up according to BCEo (categorical variable; threshold = 0.3 G/L) and maintenance therapy at each BCEo measurement. Each dot represents the hazard ratio and its 95% confidence interval of an explicative variable.

Patients with a higher eosinophil count (≥ 0.3 Giga/L) at a given follow-up time after 3 months had a 3-fold higher risk of experiencing a rejection episode during the follow-up period than patients with a lower eosinophil count (< 0.3 Giga/L) at the same time point, independent of other confounding factors (HR 2.87, 95% CI 1.38–5.98, *p* = 0.004) in patients without pre-transplant DSA and with CNI-based immunosuppression. No association was observed with BCEo ≥ 0.3 G/L when stratifying by HPR subsets (ABMR, TCMR, and borderline rejection), probably due to a lack of power since only 15 patients experienced a rejection episode 3 months post-transplant and had a BCEo ≥ 0.3 G/L (data not shown). The use of CNIs as maintenance therapy at eosinophil measurements significantly reduced the risk of a rejection episode by 76% (HR 0.24, 95% CI 0.13–0.43, *p* < 0.001), whereas the use of corticosteroids as maintenance therapy at eosinophil measurements was significantly associated with an increased risk of rejection (HR 1.82, 95% CI 1.06–3.11, *p* = 0.03) probably due that patients at higher risk of rejection (graft rank ≥ 2, pre-transplant HLA immunization) are more frequently under long-term OCS course compared to other KTR where corticosteroids withdrawal occurs early in our practice (Fig. 2 & Table S2). We found no association between BCEo and graft failure after adjusting for confounding factors (Table S3) and also between BCEo and the severity of HPR according to Banff score (Table S4). At last, we did not identify any effect of treatment rejection on BCEo duing follow-up (Fig. S2).

### 3.3 Non-statistically significant relationship between the follow-up eosinophil count and the appearance of DSAdn

**Fig. 3:**
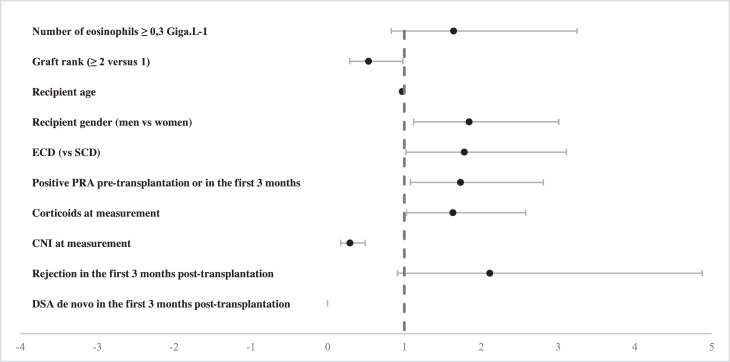
Forest plot of the time-dependent multivariable analysis of *do novo* DSA appearance (*n* = 98) in KTRs (*n* = 1013) during follow-up according to BCEo (categorical variable; threshold = 0.3 G/L) and maintenance therapy at each BCEo measurement. Each dot represents the hazard ratio and its 95% confidence interval of an explicative variable.

Non-statistically significant association was found between BCEo and the development of DSAdn during the follow-up period (HR 1.64, 95% CI 0.83–3.25, *p* = 0.15) suggesting that BCEo could be associated with DSAdn appearance in a specific subpopulation of KTR. Nevertheless, the use of CNIs as maintenance therapy at eosinophil measurements significantly reduced the risk of developing DSAdn by 71% (HR 0.29, 95% CI 0.17–0.49, *p* < 0.001), whereas the use of corticosteroids as maintenance therapy at eosinophil measurements increased the risk of DSAdn appearance (HR 1.63, 95% CI 1.03–2.58, *p* = 0.04). Not surprisingly, HLA antibody positivity before transplantation or 3 months post-transplant and extended criteria donors were significantly associated with an increased risk of developing DSAdn (HR 1.73, 95% CI 1.08–2.81, *p* = 0.02; HR 1.78, 95% CI 1.02–3.11, *p* = 0.04) (Fig. 3 & Table S5).

### 3.4 Eosinophil infiltrate was not found in kidney transplant biopsies

We found no difference in HPR subsets (ABMR, TCMR, and borderline rejection) in KTRs with a high eosinophil count compared to those with a low eosinophil count. Within the rejection group, we matched KTRs with a high eosinophil count (*n* = 15) to KTRs with a low eosinophil count (*n* = 13) by age (+/- 5 years) and sex to take into account immunosenescence. We did not detect the presence of eosinophils in the graft and thus no correlation with the blood eosinophil count in either subgroup (high or low eosinophil count) (Table 3).

**Table 3:** Characteristics of the histopathological analysis of kidney transplant biopsies in KTRs who experienced rejection and a high eosinophil count compared with KTRs who experienced rejection and a low eosinophil count. KTRs were matched for age and sex for the eosinophil infiltrate comparison.

	BCEo > 0.3G/L n = 15	BCEo < 0.3G/L n = 60	*p*-value
Subset of BPR			
TCMR (%)	33	42	0.86
ABMR (%)	47	37	0.17
Borderline (%)	20	21	1

	BCEo > 0.3 G/L *n* = 15	BCEo < 0.3 G/L *n* = 13	*p*-value

Eosinophil infiltrate (% of surface)	-	-	-
Neutrophil infiltrate (% of surface)	-	-	-
Basophil infiltrate (% of surface)	-	-	-

## 4 Discussion

First described in 1879 by Ehrlich [33] because of their capacity to be stained by acidophilic dyes, the function of eosinophils in health and diseases and particularly their role and presence in the blood remain elusive. Indeed, eosinophils in the blood represent only very few cells (< 0.5 G/L), and before they migrate into the thymus, they spend little time in the peripheral blood, with a half-life of approximately 18 h [34].

Eosinophils are mainly associated with type 2 inflammatory diseases but have also been described in organ transplantation, mainly in the lung and liver. In lung transplantation, a BCEo ≥ 0.4 G/L, though arbitrary, is used as a biological sign suggesting acute lung rejection in the presence of a 10% FEV1 decrease during patient follow-up [35]. To our knowledge, only two retrospective studies have evaluated the link between BCEo and pulmonary graft rejection in lung transplant recipients (LTRs) [17,36]. Trull et al. observed that the mean absolute BCEo (0.14 G/L) was higher within five days before rejection and that an increase of 0.09 G/L in BCEo in allograft recipients allowed the detection of acute AR one month post surgery [17]. On the other hand, Kaes et al. recently observed that a relative BCEo ≥ 8% in LTRs during follow-up significantly increased the risk of chronic allograft dysfunction by 67% independent of known eosinophilia-related confounding factors such as initial lung disease, antibiotic use (mostly meropenem) and allograft infection at the eosinophil peak [36]. However, in both studies, the effect of BCEo was not adjusted for the systematic use of oral corticoids as maintenance therapy, whereas inverse linear correlation exists with BCEo [37]. Moreover, LTRs are frequently leukopenic due to the triple immunosuppressive regimen (a CNI + an antiproliferative and corticoids), rending it difficult to compare the relative BCEo from one patient to another. Finally, there is circadian intraindividual variation in eosinophilia up to 20%, with a peak at approximately midnight [30,31,38]. Thus, it becomes difficult or even impossible to discriminate a BCEo variation of 0.09 G/L as proposed by Trull et al. [17] as physiologic or related to acute rejection within 3 months post operation.

In liver transplantation, BCEo has long been associated with acute cellular rejection [39]. Two studies demonstrated that an elevated absolute BCEo ≥ 0.4 G/L within five days before HPR was likely associated with the severity of acute cellular rejection in the postoperative period [40,41]. Nevertheless, one major limitation of those studies was the immunosuppressive regimen with a low dose of a CNI (compared to kidney and lung transplantation) and the decreasing dose of systemic corticoids in the postoperative period, suggesting that the increase in BCEo could also be a collateral effect of IS drug modulation [37,42], as confirmed by its poor sensitivity (approximately 30%) in predicting HPR.

Despite being less frequent, several studies also reported the appearance of eosinophils in the peripheral blood [13,14,[43], [44], [45]] and kidney transplant biopsies [43,[46], [47], [48], [49], [50]]. Nevertheless, the prognostic significance of these eosinophils regarding kidney rejection has not been elucidated and remains a debated question. Interestingly, we demonstrated in a cohort of 1013 KTRs a significant association between time-dependent variation in BCEo and the onset of an immunological event and, more precisely, the onset of HPR after 3 months post-transplant independent of the immunosuppressive regimen in patients without pre-transplant DSA and with CNI-based immunosuppression. Patients with an absolute BCEo threshold ≥ 0.3 G/L are thus associated with an increased risk of an immunological outcome. Two previous studies on kidney transplantation reported that a relative BCEo ≥ 4% at the time of graft biopsy was associated with acute rejection, and these studies also reported an association with poor graft survival [13,14]. These data are concordant with ours regarding the impact on rejection since an absolute BCEo threshold ≥ 0.3 G/L represents a BCEo of approximately 4% considering a total leukocyte count of approximately 7 G/L. Nevertheless, we found that BCEo was associated neither with DSAdn appearance nor with graft survival. These discrepancies may be because the previous studies were conducted within 2 months post kidney transplantation, a period that is highly impacted by induction therapies such as steroid pulses and/or depletion therapy. Thus, there may have been an overestimation of the relative BCEo (higher percentage for the same absolute BCEo) in patients with leukopenia and/or lymphopenia since the majority of patients received depletion therapy (antilymphocyte serum or CD3 antagonist monoclonal antibodies) at induction, which was not the case in our study. Furthermore, CNI monotherapy, which was the most frequent maintenance immunosuppressive regimen in those studies, has been shown to be associated with a high absolute and relative BCEo [42,46]. In our study, patients were treated with an IS regimen comprising a CNI and an antiproliferative +/- OCS (Table 1).

Many confounding factors (postoperative period, induction therapy and maintenance IS regimen, and circadian and intra-individual variability) could impact the relative and absolute BCEo thresholds, thus questioning their robustness in predicting HPR in solid organ transplantation. Importantly, steroids are clearly one of the main factors that downregulate eosinophils [32]. The strength of our cause-specific model was that BCEo and IS drugs were considered at each measurement in a time-dependent manner and thus contributed to overcome the uncertainties about the impact of CNI and oral corticoids association on BCEo during follow-up. Of important note, none of the KTRs with HPR with a BCEo > 0.3 G/L had skin and/or cutaneous and/or gastro-intestinal symptoms in their medical history that could suggest an add-on eosinophilic disorder [51].

In the literature, the results regarding a correlation between the number of eosinophils in the graft and in the blood are controversial. Whereas some report a direct correlation [14,43,49], other report a smaller or larger number in the graft [50,52] according to the method of detection. We did not detect an association between eosinophilic infiltrate and an increase in blood eosinophilia in our cohort of patients. This is not surprising since in the literature, biopsy eosinophilia is associated with acute rejection occurring within the first 2 months [13,14,43,50], whereas our follow-up began 3 months post-transplant, suggesting a difference in eosinophilia immunopathology between the postoperative period and steady state (3 months post-transplant). A hypothesis is that eosinophils in the graft could result from ischemia/reperfusion damage (DAMP production, such as ATP/ADP and local metabolic changes), stimulating and polarizing the innate immune response toward type 2 immunity [1,53]. Furthermore, eosinophil infiltrates were observed in patients treated with CsA monotherapy [13,14,43], which was not the case in our study, in which patients were treated with a CNI and an antiproliferative +/- OCS (Table 1).

The question is then “What could trigger eosinophilia?” and, by extension, “What could trigger type 2 immunity 3 months post kidney transplantation?”, particularly in the blood. Contrary to common paradigms, type 2 immunity is not limited to allergic, parasitic or fungal hypersensitivity. Recently, specific stimuli in the cell microenvironment were shown to strongly polarize innate immunity to a type 2 response (secretion of IL-33, TSLP, IL-25, RANTES, CCL11 [aka eotaxin-1], IL-4, IL-13, and IL-5). These stimuli act alone or in synergy and consist of either protease-activated receptors (PARs) via the serine/cysteine protease activity of antigens and/or tissue damage release of DAMPs and/or metabolic changes leading to the oxidative stress response (*e.g.*, amino acid starvation or a decrease in partial pressure in oxygen) [1]. Several questions/hypotheses arise from these concepts. As mentioned above, tissue damage during ischemia/reperfusion may explain eosinophilia and eosinophil infiltration of the transplant in the early months post-transplant [54]. However, it cannot explain eosinophilia at steady state. An interesting hypothesis relies on PAR activation by tryptase, a neutral cysteine protease produced exclusively by tissular mast cells. Indeed, tryptase-induced PAR-2 activation was shown to mediate eosinophil activation/recruitment and to induce IL-13 secretion, thus amplifying type 2 inflammation [55], [56], [57]. Furthermore, it was recently demonstrated that DSA-IgE could be detected in the blood of KTRs, and IgE deposits colocalizing with mast cells were found in patients who experienced ABMR [5,58]. Unfortunately, we did not collect serum samples around BCEo≥ 0.3 G/L in our cohort to titrate IL-5, IL-4, IL13 and/or CCL-11 to test this hypothesis. Another interesting hypothesis, though more uncertain, could be viral infection by Herpesviridae (mostly EBV and CMV), which is associated with an increased risk of long-term transplant loss [59,60]. Indeed, eosinophils are associated with EBV-induced Hodgkin lymphoma, and active viral infection is usually associated with oxidative stress [61,62]. LMP-1, which is an EBV major inflammatory protein, can upregulate IgE production [63]. However, we did not have concomitant PCR reports in patients with high levels of eosinophils in our study. Finally, one study reported a strong correlation between recipients with HLA-B8 and an increased risk of eosinophilia [50], which was not the case in our study. Indeed, HLA-B8 was found in 13,8% of KTRs who experienced immunological events, 12% of KTRs who experienced rejection and 13% of KTRs who did not experience any immunological event (data not shown). Altogether, these data suggest that time-dependent eosinophilia is associated with immunological events and, more precisely, that transplant rejection could reflect (1) a humoral IgE response against the graft (DSA-IgE) and consecutive mast cell activation and (2) active viral infection (EBV, CMV) that activates/amplifies type 2 immunity against kidney transplantation. Though, it should be born in mind that recent studies have highlighted an immune-regulatory role of eosinophils through different mechanisms such as galectin 10 in a model of graft versus host disease [64], PD-L1 expression in response to stimulation by INF-*γ* or by the iNOS pathway in a mice model of lung transplantation [65]. Altogether, it suggests that eosinophils could be either deleterious or protective in solid organ transplantation likely due to micro-environment but also the duration of the stimuli (acute versus chronic inflammation). Further mechanistic research are needed to better understand the meaning of the association that our results have highlighted.

Our study presents some limitations due to its experimental design and its bio-statistical modelling. Indeed, limitations remain the absence of a precise time point associated with a BCEo ≥ 0.3 G/L and an immunological outcome and its monocentric design. Furthermore, confounding factors not recorded in our data base such as time of sampling, exercise or food intake before sampling can decrease BCEo [31] leading to a potential under-estimation of its impact on immune event outcome. Another limit of our study is the insufficient control of exposure to CNIs or corticoids and their impacts on BCEo during follow-up that we could roughly handle by considering CNI and corticoids as time dependent variable at each BCEo measurement. A more accurate impact of those immunosuppressive drug could have been achieved by considering the cumulative exposure in our model (unavailable for corticoids). At last, BCEo time dependent variations do not strictly correlate with eosinophils infiltration or tissue-injuries all the more so patients are under immunosuppressive drugs [66,67]. Thus, BCEo cannot be considered as a predictive biomarker of rejection on its own. At last, our study does not explore any physiopathological mechanisms of eosinophil on kidney transplant rejection although some hypotheses were discussed for further mechanistic researches.

## 5 Conclusion

Our data thus revealed that a BCEo ≥ 0.3 G/L threshold could be an interesting and routine biological marker for monitoring immunological outcomes along with other routine parameter such as DSAdn in kidney transplant recipients without pre-transplant DSAs and with CNI-based immunosuppression at steady state (*i.e.*, 3 months post-transplant). In clinical practice after eliminating common causes of an increase in BCEo (PTLD, acute allergic process, parasitic or viral infections) [19], BCEo ≥ 0.3 G/L could lead to monitor more regularly biological parameters associated with rejection such as DSAdn (IgG anti-HLA class I or II with MFI >2000) and/or proteinuria > 1 g/24 h and/or hematuria (> 10 red blood cells/mL) and/or an increase of 25% in serum creatinine compared to baseline. Though, multicentric studies challenging BCEo > 0.3 G/L threshold but also evaluating the optimal time points of BCEo titration are needed. At last, these observations open new perspectives and directions that raise the question of the involvement of eosinophils and type 2 immunity in kidney allograft rejection.

## Contributors

LC conceptualized, created the methodology, curated data and formal analysis, wrote the original draft, reviewed, and edited. LB created the methodology, curated data and formal analysis. CK managed the data and reviewed the manuscript. KAR, ML and AM reviewed and edited the manuscript. MG conceptualized, created the methodology, reviewed, and edited the manuscript. SB conceptualized, created the methodology, curated datas, wrote, reviewed, and edited the manuscript. All the authors red and approved this manuscript.

## Divat cohort collaborators list

Pr. Gilles Blancho; Dr. Julien Branchereau; Dr. Diego Cantarovich; Dr. Anne Cesbron; Dr. Agnès Chapelet; Pr. Jacques Dantal; Dr. Florent Delbos; Dr. Clément Deltombe; Dr. Anne Devis; Dr. Lucile Figueres; Dr. Claire Garandeau; Dr. Caroline Gourraud-Vercel; Pr. Maryvonne Hourmant; Dr. Christine Kandell; Pr. Georges Karam; Dr. Aurélie Meurette; Dr. Anne Moreau; Dr. Simon Ville; Dr. Alexandre Walencik.

## Declaration of Competing Interest

The authors of this manuscript have no conflicts of interest to disclose as described by EBioMedicine.

## Appendix Supplementary materials

Image, application 1
