# Supplementary material for: Time-dependent blood eosinophilia count increases the risk of kidney allograft rejection
Source: eBioMedicine. 2021 Oct 20;73:103645. doi: 10.1016/j.ebiom.2021.103645 (PMC8536518; doi:10.1016/j.ebiom.2021.103645)
Supplement: Supplementary file 1 [file mmc1.docx]

Supplementary datas:

Figure S1. Statistical analysis confirming the a priori BCEo >0.3G/L threshold could be appropriate to model the main outcome of the study. (A) represent the Martingale residuals according to continuous BCEo. (B) represent Spline function of blood eosinophil according to hazarad ratio using cause-specific multivariable Cox model.

*A.*

*
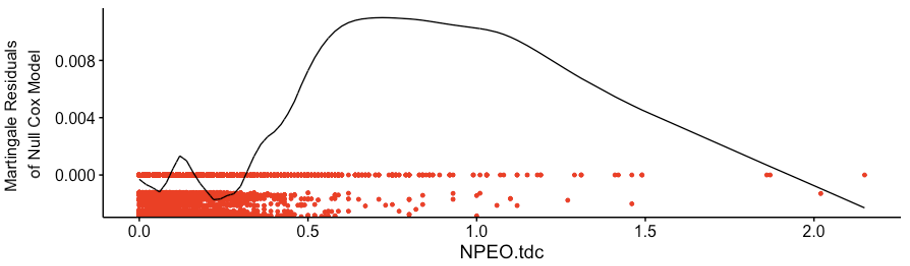
*

*B.*

*
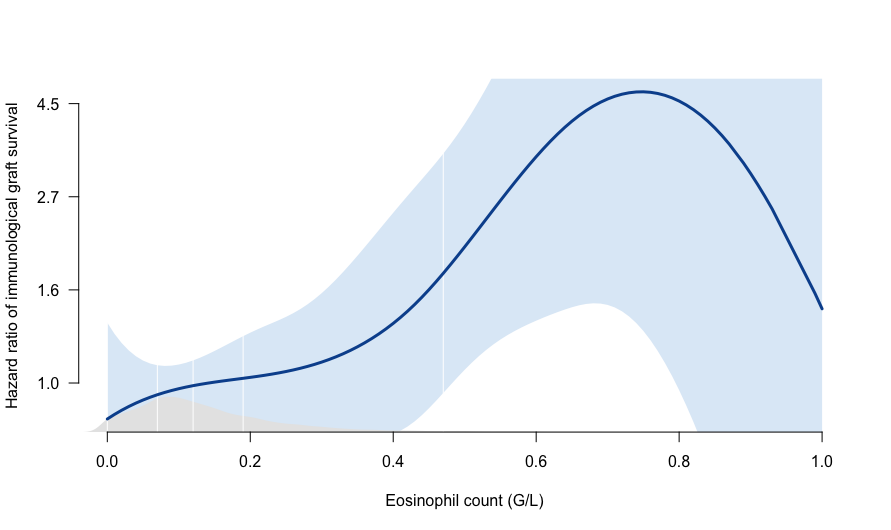
*

*Figure S2.* BCEo evolution in KTR experiencing HPR according to their rejection treatment.


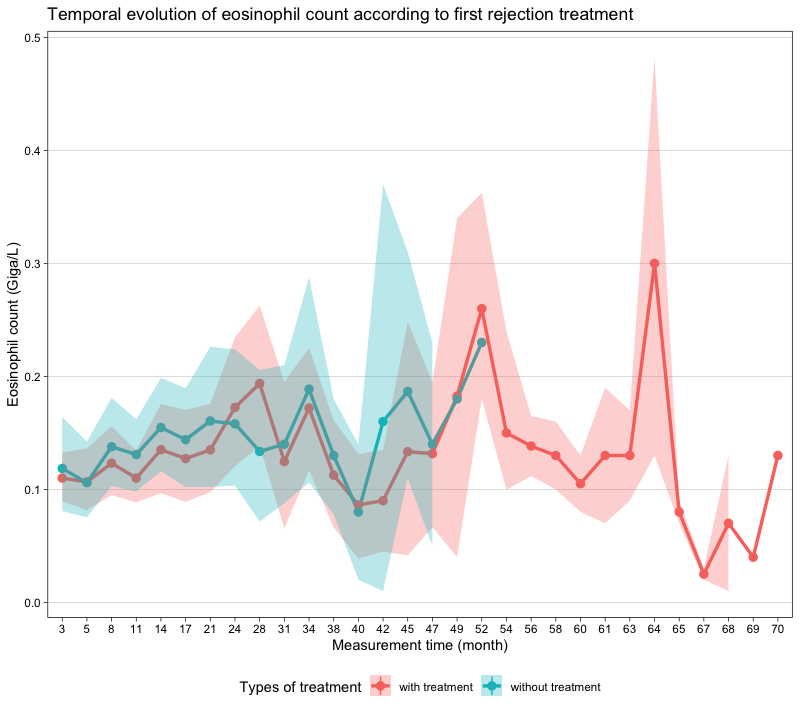


*Table S1*: Time-dependent multivariable analysis of immunological event onset (rejection and/or DSA appearance; n=149) in KTRs (n=1013) during follow-up according to BCEo (categorical variable) and maintenance therapy at each BCEo measurement. HR = Hazard ratio; 95% CI = 95% Confidence interval.

|  | **HR** | **95% CI** | **p-value** |
| --- | --- | --- | --- |
| Number of eosinophils between 0.1-0,2 Giga.L^-1^ | 0.72 | 0.92-2.06 | 0.12 |
| Number of eosinophils between 0.2-0,3 Giga.L^-1^ | 1.19 | 0.43-1.60 | 0.59 |
| Number of eosinophils ≥ 0,3 Giga.L^-1^ | 2.52 | 1.23-4.11 | 0.008 |
| Graft rank (≥ 2 versus 1) | 0.68 | 0.41-1.12 | 0.13 |
| Recipient age | 0.97 | 0.95-0.99 | 0.0009 |
| Recipient gender (men vs women) | 1.30 | 0.88-1.91 | 0.17 |
| ECD (vs SCD) | 1.43 | 0.91-2.25 | 0.12 |
| Positive Panel-Reactive Antibody pre-transplantation or in the first 3 month | 1.18 | 0.80-1.76 | 0.41 |
| Corticoids at measurement | 1.72 | 1.19-2.51 | 0.004 |
| CNI at measurement | 0.30 | 0.19-0.45 | <0.001 |
| Rejection in the first three months post-transplantation | 2.01 | 1.04-3.90 | 0.04 |
| DSA de novo in the first three months post-transplantation | 0.65 | 0.20-2.16 | 0.49 |

*Table S2*. Time-dependent multivariable analysis of the first kidney allograft rejection episode (n= 75) in KTRs (n=1013) during follow-up according to BCEo (categorical variable; threshold = 0.3 G/L) and maintenance therapy at each BCEo measurement. HR = Hazard ratio; 95% CI = 95% Confidence interval.

|  | **HR** | **95% CI** | **p-value** |
| --- | --- | --- | --- |
| Number of eosinophils ≥ 0,3 Giga.L^-1^ | 2.87 | 1.38-5.98 | 0.004 |
| Graft rank (≥ 2 versus 1) | 0.81 | 0.37-1.73 | 0.58 |
| Recipient age | 0.97 | 0.95-0.99 | 0.01 |
| Recipient gender (men vs women) | 0.77 | 0.46-1.31 | 0.38 |
| ECD (vs SCD) | 1.74 | 0.91-3.32 | 0.09 |
| Positive Panel-Reactive Antibody pre-transplantation or in the first 3 month | 0.55 | 0.30-1.01 | 0.054 |
| Corticoids at measurement | 1.82 | 1.06-3.11 | 0.03 |
| CNI at measurement | 0.24 | 0.13-0.43 | <0.001 |
| Rejection in the first three months post-transplantation | 2.24 | 0.94-5.37 | 0.07 |
| DSA de novo in the first three months post-transplantation | 1.63 | 0.46-5.74 | 0.45 |

*Table S3*. Time-dependent multivariable analysis of graft failure in KTRs (n=1013) during follow-up according to BCEo (categorical variable; threshold = 0.3 G/L) and maintenance therapy at each BCEo measurement. HR = Hazard ratio; 95% CI = 95% Confidence interval.

|  | **HR** | **95% CI** | **p-value** |
| --- | --- | --- | --- |
| Number of eosinophils ≥ 0,3 Giga.L^-1^ | 1.72 | 0.93-3.21 | 0.09 |
| Graft rank (≥ 2 versus 1) | 0.83 | 0.50-1.40 | 0.49 |
| Recipient age | 0.99 | 0.97-1.00 | 0.11 |
| Recipient gender (men vs women) | 1.08 | 0.70-1.65 | 0.80 |
| ECD (vs SCD) | 0.61 | 0.37-1.03 | 0.06 |
| Positive Panel-Reactive Antibody pre-transplantation or in the first 3 month | 1.18 | 0.74-1.88 | 0.49 |
| Corticoids at measurement | 3.47 | 2.23-5.39 | <0.001 |
| CNI at measurement | 0.40 | 0.25-0.64 | <0.001 |
| Rejection in the first three months post-transplantation | 2.38 | 1.23-4.63 | 0.01 |
| DSA de novo in the first three months post-transplantation | 1.36 | 0.52-3.60 | 0.53 |

*Table S4.* Table S4. Association between severity of HPR according to Banff items and the lats BCEo before rejection.

|  | **Correlation coefficient** | **Standard error** | **p-value** |
| --- | --- | --- | --- |
| Presence of C4d | -0.02 | 0.05 | 0.66 |
| Glomerulitis (g) | -0.02 | 0.02 | 0.39 |
| Interstitial inflammation (i) | -0.03 | 0.02 | 0.21 |
| Tubulitis (t) | 0.03 | 0.02 | 0.26 |
| Vasculitis (v) | 0.07 | 0.04 | 0.12 |
| (cpt) | 0.02 | 0.02 | 0.38 |
| (cg) | 0.03 | 0.02 | 0.14 |

*Table S5*: Time-dependent multivariate analysis of *do novo* DSA appearance (n= 98) in KTRs (n=1013) during follow-up according to BCEo (categorical variable; threshold = 0.3 G/L) and maintenance therapy at each BCEo measurement. HR = Hazard ratio; 95% CI = 95% Confidence interval.

|  | **HR** | **95% CI** | **p-value** |
| --- | --- | --- | --- |
| Number of eosinophils ≥ 0,3 Giga.L^-1^ | 1.64 | 0.83-3.25 | 0.15 |
| Graft rank (≥ 2 versus 1) | 0.53 | 0.29-0.98 | 0.04 |
| Recipient age | 0.97 | 0.95-0.98 | <0.001 |
| Recipient gender (men vs women) | 1.84 | 1.12-3.01 | 0.02 |
| ECD (vs SCD) | 1.78 | 1.02-3.11 | 0.04 |
| Positive Panel-Reactive Antibody pre-transplantation or in the first 3 month | 1.73 | 1.08-2.81 | 0.02 |
| Corticoids at measurement | 1.63 | 1.03-2.58 | 0.04 |
| CNI at measurement | 0.29 | 0.17-0.49 | <0.001 |
| Rejection in the first three months post-transplantation | 2.11 | 0.91-4.88 | 0.08 |
| DSA de novo in the first three months post-transplantation | - | - | 0.99 |
